# Supplementary material for: Targeting of early endosomes by autophagy facilitates EGFR recycling and signalling
Source: EMBO Rep. 2019 Aug 26;20(10):e47734. doi: 10.15252/embr.201947734 (PMC6776898; doi:10.15252/embr.201947734)
Supplement: Supplementary file 1 — Expanded View Figures PDF [file EMBR-20-e47734-s001.pdf]

Expanded View Figures

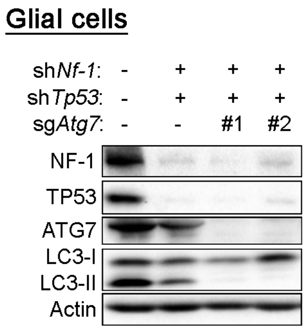

Figure EV1. Confirmation of CRISPR/Cas9-mediated gene editing and autophagy inhibition in cells.

Western blot analyses of glial cells infected with shRNA against *Nf-1* and *Tp53*, and then with Cas9 and sgRNAs against *Atg7*.

© 2019 The Authors

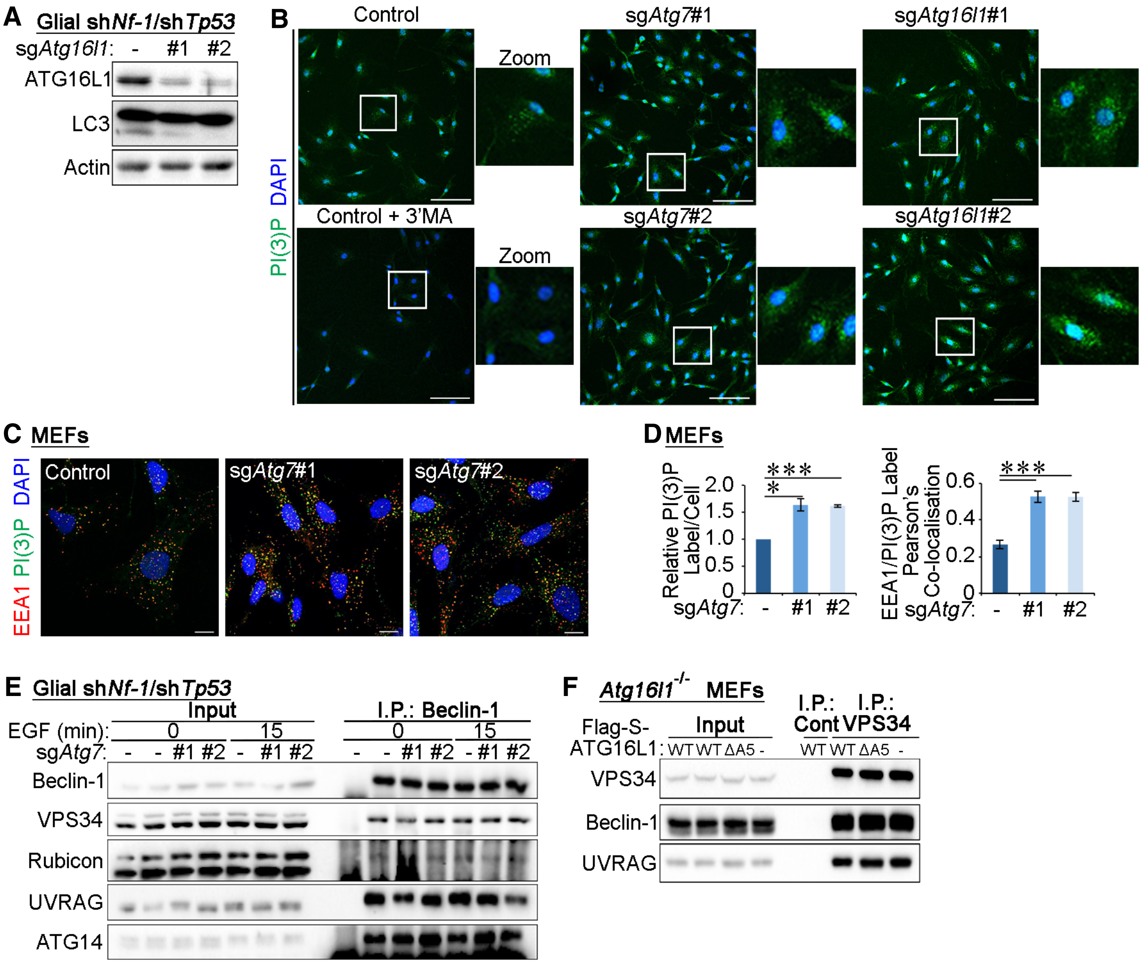

Figure EV2. Total PI(3)P levels increase in autophagy-inhibited cells.

All cells were serum starved for 4 h before assaying.

- A Western blot analyses of sh*Nf-1*/sh*Tp53* glial cells expressing gRNA sequences targeting *Atg16l1*.
- B 20× magnification of images in Fig 2A. Control, sg*Atg7*, or sg*Atg16l1* cells were treated with 2 ng/ml EGF for 15 min. Cells were then processed for staining using a PI(3)P probe (Alexa 488-labelled 2XFYVE domain). To ensure the specificity of the probe, control cells were pre-treated with 5 mM 3'MA for 30 min. Scale bar: 100 μm.
- C Control or sg*Atg7* MEF cells were treated with 2 ng/ml EGF for 15 min before fixation and staining using EEA1 antibodies and a PI(3)P probe (Alexa 488-labelled 2XFYVE domains). Scale bar: 10 μm.
- D Quantification of PI(3)P^{+} vesicles per cell and the Pearson's colocalisation coefficient between PI(3)P and EEA1 (in C).
- E Endogenous Beclin-1 was immunoprecipitated from control and sg*Atg7* cells that were stimulated with 2 ng/ml EGF for 15 min. Cells were lysed in CHAPS buffer followed by immunoprecipitation of Beclin-1 and analyses by Western blotting.
- F *Atg16l1*$^{-/-}$ MEFs were reconstituted with wild type (WT) ATG16L1 or a mutant of ATG16L1 containing a deletion in the ATG5 binding domain (residues 1–39, ΔA5). Endogenous VPS34 was immunoprecipitated from these cells following 15 min of EGF (2 ng/ml). Binding partners were assessed by Western blotting.

Data information: Statistical analyses were performed on at least three independent experiments, where error bars represent SEM and *P* values represent a two-tailed Student's *t*-test: *$P < 0.05$, ***$P < 0.001$.

Figure EV3. The canonical autophagy machinery is required for early endosome targeting.

A MEF cells stably expressing GFP-LC3 were serum starved for 4 h, then treated with 100 μM monensin for 1 h and stimulated with 2 ng/ml EGF for 15 min before immunofluorescence staining against EEA1. White arrows indicate colocalisation. Scale bar: 10 μm.

B Quantification of percentage of total EEA1 vesicles that colocalise with GFP-LC3 (in A).

C Box-and-whisker representation of the quantification of the number of EEA1-positive vesicles per cell in control and sg*Atg7* cell lines. No significant differences are observed in early endosome numbers between control and autophagy-inhibited cells (control versus sg*Atg7*#2: P = 0.29, control versus sg*Atg7*#3: P = 0.43).

D Glial sh*Nf-1*/sh*Tp53* control cells stably expressing Flag-S-ATG16L1 were either serum starved (-FBS) or treated with amino acid-free DMEM (-AA) for 4 h before immunofluorescence staining against Flag tag and EEA1. Scale bar: 10 μm.

E Glial sh*Nf-1*/sh*Tp53* control and sg*Atg7* cells stably expressing wild-type ATG16L1 (Flag-S-ATG16L1^{WT}) or LAP-deficient K490A mutant of ATG16L1 (Flag-S-ATG16L1^{K490A}) were serum starved for 4 h followed by stimulation with 2 ng/ml EGF for 15 min and immunofluorescence staining to detect Flag tag and EEA1. Scale bar: 10 μm.

F Quantification of percentage of total EEA1 vesicles positive for Flag-S-ATG16L1 (in E) with quantification of endogenous ATG16L1 included (see Fig 4E).

G *Atg16l1*$^{-/-}$ MEF cells stably expressing wild-type (Flag-S-ATG16L1^{WT}) or LAP-deficient mutant (Flag-S-ATG16L1^{K490A}) of ATG16L1 were serum starved for 4 h followed by stimulation with 2 ng/ml EGF for 15 min and immunofluorescence staining to detect Flag tag and EEA1. White arrows indicate colocalisation. Scale bar: 10 μm.

H Quantification of the percentage of total EEA1 vesicles that stain positive for wild-type or the K490A point mutant of ATG16L1 expressed in *Atg16l1*$^{-/-}$ MEFs (in G).

I *Atg13*$^{-/-}$ MEF cells were treated for 1 h with 100 μM monensin and then stimulated with 2 ng/ml EGF for 15 min. ATG16L1 and EEA1 were then detected by immunofluorescence staining. White arrows indicate colocalisation. Scale bar: 10 μm.

J Quantification of percentage of total EEA1 vesicles positive for Flag-S-ATG16L1 (in I).

Data information: Statistical analyses were performed on at least three independent experiments, where error bars represent SEM and P values represent a two-tailed Student's t-test: NS $P > 0.05$, *$P < 0.05$, **$P < 0.01$, ***$P < 0.001$.

© 2019 The Authors

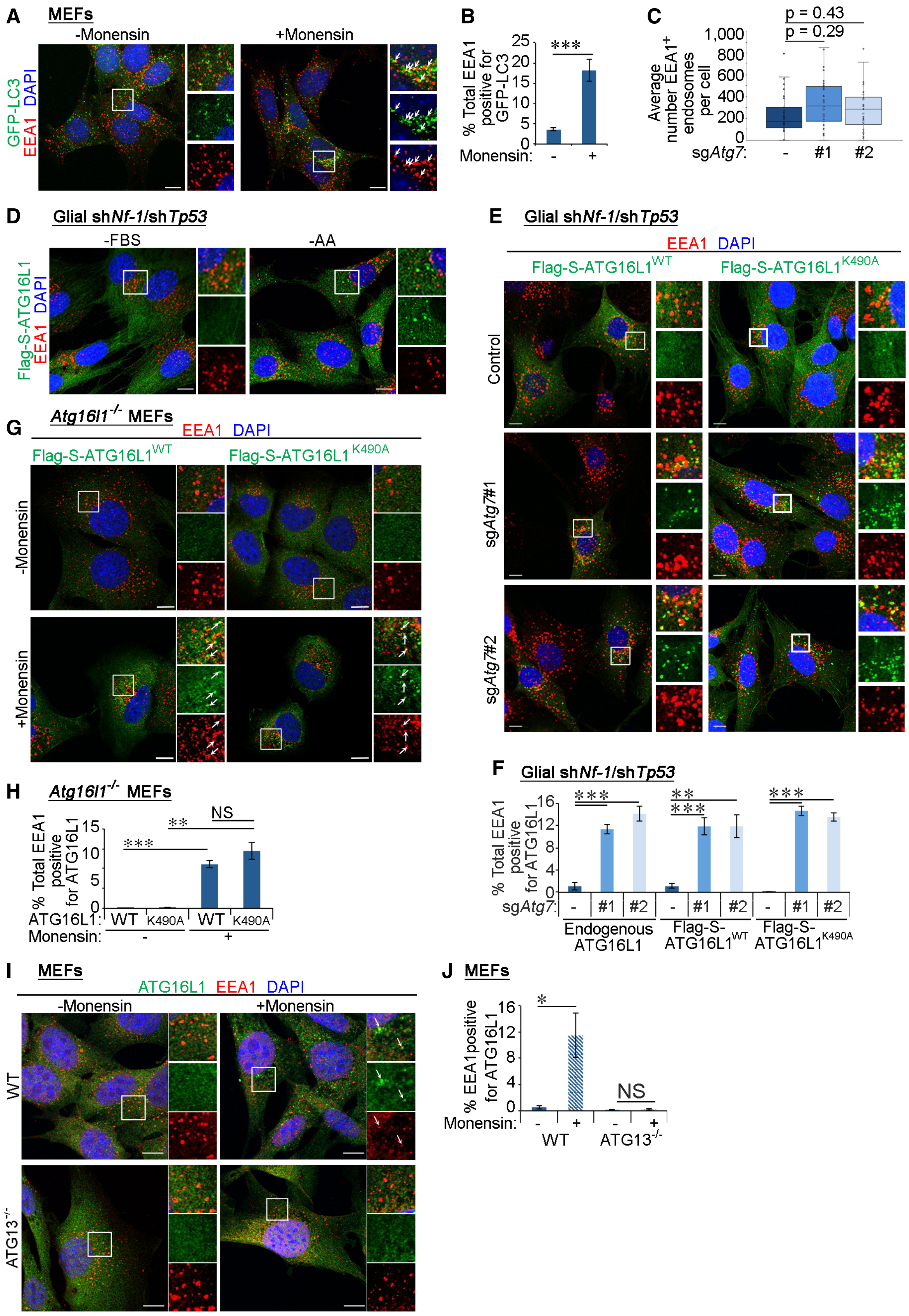
A MEFs
-Monensin +Monensin
GFP-LC3 EEA1 DAPI
B
% Total EEA1 positive for GFP-LC3
***
Monensin: - +
C
p = 0.43
p = 0.29
Average number EEA1+ endosomes per cell
sgAtg7: - #1 #2
D Glial shNf-1/shTp53
-FBS -AA
Flag-S-ATG16L1 EEA1 DAPI
E Glial shNf-1/shTp53
EEA1 DAPI
Flag-S-ATG16L1WT Flag-S-ATG16L1K490A
Control
sgAtg7#1
sgAtg7#2
G Atg16l1-/- MEFs
EEA1 DAPI
Flag-S-ATG16L1WT Flag-S-ATG16L1K490A
-Monensin
+Monensin
H Atg16l1-/- MEFs
% Total EEA1 positive for ATG16L1
*** ** NS
ATG16L1: WT K490A WT K490A
Monensin: - +
F Glial shNf-1/shTp53
% Total EEA1 positive for ATG16L1
*** ** *** ***
sgAtg7: - #1 #2 - #1 #2 - #1 #2
Endogenous ATG16L1
Flag-S-ATG16L1WT
Flag-S-ATG16L1K490A
I MEFs
ATG16L1 EEA1 DAPI
-Monensin +Monensin
WT
ATG13-/-
J MEFs
% EEA1positive for ATG16L1
* NS
Monensin: - + - +
WT ATG13-/-

Figure EV3.

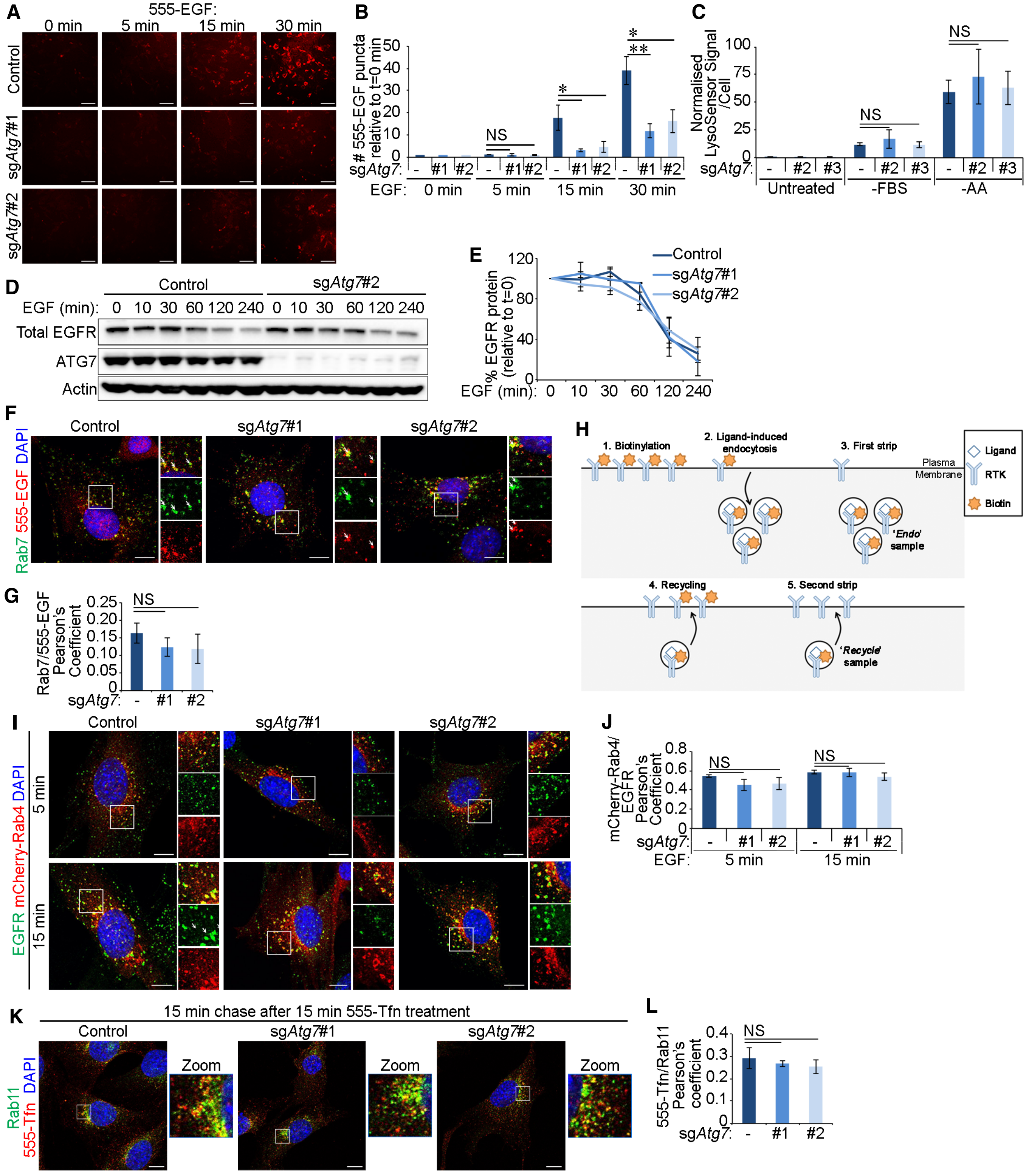

Figure EV4.

 © 2019 The Authors

Figure EV4. Targeting of EGFR to late endosomes and Rab4^{+} recycling endosomes remain intact in the absence of autophagy.

The following experiments were performed in sh*Nf-1*/sh*Tp53* glial cells serum starved for 4 h before assaying. Cells were expressing either Cas9 alone (control) or Cas9 and sgRNA targeting *Atg7* (sg*Atg7* #1 or #2).

A Cells were treated with 20 ng/ml Alexa 555-EGF (555-EGF) for the indicated times before fixation. 555-EGF was detected using the ImageXpress high-content imaging platform, and representative images of 555-EGF are shown. Scale bar: 100 µm.

B Following high-content imaging of 555-EGF and DAPI, 555-EGF uptake relative to cell number was quantified and made relative in each cell line to background readings from unstimulated cells (t = 0 min) (in A).

C Control or sg*Atg7* glial sh*Nf-1*/sh*Tp53* cells were either untreated, serum starved (-FBS) for 4 h, or amino acid starved (-AA) for 2 h and LysoSensor probe added for 1 h. Shown are quantifications of LysoSensor fluorescence intensity per cell normalised to untreated control cells.

D Cells were stimulated with 20 ng/ml EGF for the indicated times before EGFR levels were analysed by Western blotting.

E Densitometry analyses of Western blotting showing EGFR degradation with measurements made relative to EGFR levels in unstimulated cells (t = 0 min) (in D).

F Cells were stimulated with 20 ng/ml 555-EGF for 15 min before immunofluorescence staining against endogenous Rab7. White arrows indicate colocalisation. Scale bar: 10 µm.

G Quantification of Pearson's colocalisation coefficient between 555-EGF and Rab7 (in F).

H Schematic representation of cell surface protein biotinylation assay to assess endocytosis and recycling rates (see Fig 6C and D).

I Cells stably expressing mCherry-Rab4 were stimulated with 20 ng/ml EGF for 5 or 15 min and then fixed and stained by immunofluorescence against EGFR. White arrows indicate colocalisation. Scale bar: 10 µm.

J Quantification of Pearson's colocalisation coefficient between mCherry-Rab4 and EGFR (in I).

K Cells were stimulated with Alexa 555-transferrin (555-Tfn, 20 ng/ml) for 15 min and chased with media without transferrin for 15 min. Cells were then stained by immunofluorescence against Rab11. Scale bar: 10 µm.

L Quantification of Pearson's colocalisation coefficient between 555-Tfn and Rab11 (in K).

Data information: Statistical analyses were performed on at least three independent experiments, where error bars represent SEM and P values represent a two-tailed Student's t-test: NS $P > 0.05$, *$P < 0.05$, **$P < 0.01$.

© 2019 The Authors

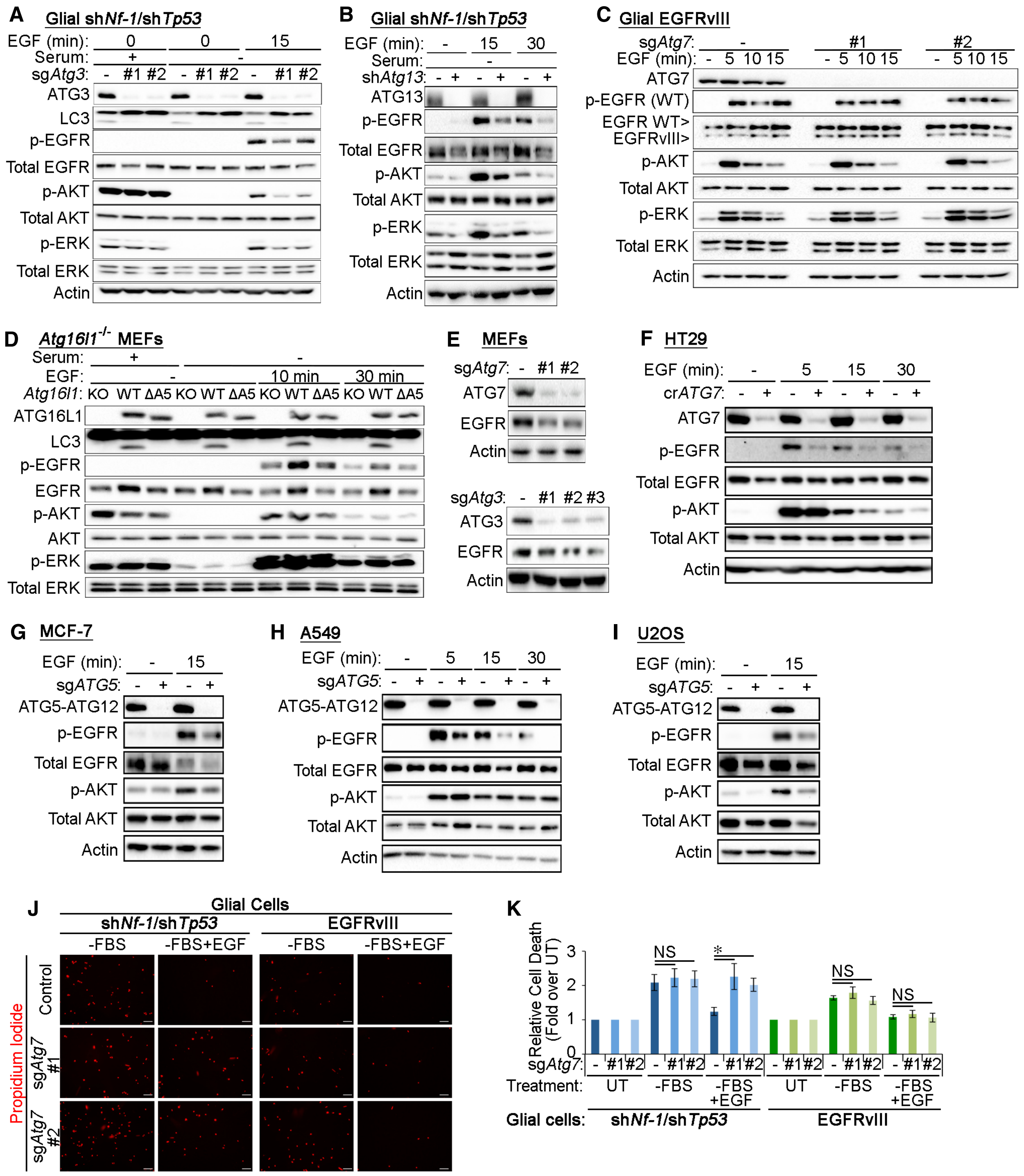

Figure EV5.

 © 2019 The Authors

Figure EV5. Analyses of EGFR signalling and levels in various cell lines upon autophagy inhibition.

A Glial sh*Nf-1*/sh*Tp53* sg*Atg3* cells were either left untreated (+serum) or serum starved for 4 h and then stimulated with 2 ng/ml EGF for 15 min before analysis by Western blotting.

B Glial sh*Nf-1*/sh*Tp53* sh*Atg13* cells were serum starved for 4 h and then stimulated with 2 ng/ml EGF for 15 or 30 min before analysis by Western blotting.

C Glial EGFRvIII-expressing control and sg*Atg7* cells were serum starved for 4 h and then stimulated with 2 ng/ml EGF before analysis by Western blotting.

D *Atg16l1*$^{-/-}$ MEFs were reconstituted with wild-type (WT) ATG16L1 or with a mutant of ATG16L1 with deletion of the ATG5-binding domain (residues 1-39, ΔA5). Cells were either untreated or serum starved for 4 h and then stimulated with 2 ng/ml EGF for 10 or 30 min before signalling pathway analyses by Western blotting.

E Total EGFR levels in untreated sg*Atg7* and sg*Atg3* MEFs were analysed by Western blotting.

F–I The following cells were serum starved for 4 h followed by stimulation with 2 ng/ml EGF before Western blot analyses: HT29 control and cr*ATG7* (F); MCF-7 control and sg*ATG5* (G); A549 control and sg*ATG5* (H); and U2OS control and sg*ATG5* (I).

J Representative images of propidium iodide staining in control or sg*Atg7* glial sh*Nf-1*/sh*Tp53* or EGFRvIII-expressing cells that were serum starved for 24 h in the presence or absence of 20 ng/ml EGF. Scale bar: 100 μm.

K Quantification of the experiment in J with propidium iodide relative fluorescence units (RFU) made relative to cell number as assessed by DAPI RFU measured by a fluorescence plate reader. These values were then made relative to the values in the negative control setting (untreated cells, UT).

Data information: Statistical analyses were performed on at least three independent experiments, where error bars represent SEM and *P* values represent a two-tailed Student's *t*-test: NS $P > 0.05$, *$P < 0.05$.

© 2019 The Authors
